# Occurrence of hypoxic liver injury in patients with acute respiratory distress syndrome: A retrospective cohort study

**DOI:** 10.1016/j.jointm.2026.02.006

**Published:** 2026-05-09

**Authors:** Fabian Gleibs, Josephine Braunsteiner, Markus Haar, Stefan Kluge, Marlene Fischer, Kevin Roedl

**Affiliations:** 1 Department of Intensive Care Medicine, University Medical Centre Hamburg-Eppendorf, Hamburg, Germany; 2 Department of Neurology, Medical University of Innsbruck, Innsbruck, Austria

**Keywords:** Acute respiratory distress syndrome, Hypoxic liver injury, Ischemic hepatitis, Hypoxic hepatitis, Liver failure, Critically ill

## Abstract

**Background:**

Hypoxic liver injury (HLI) is a frequent and life-threatening complication occurring in up to 10% of critically ill patients. HLI can be typically found in a setting of cardiac, circulatory, or respiratory failure. Data regarding the occurrence, course, and outcome of HLI in acute respiratory distress syndrome (ARDS) is scarce. This study aimed to investigate the incidence, clinical characteristics, and outcome of HLI in critically ill, mechanically ventilated patients with ARDS.

**Methods:**

This is a single-center, retrospective observational study conducted at the intensive care units (ICUs) of a tertiary-care university hospital in Hamburg, Germany from December 1, 2017 to April 30, 2021. It includes all consecutive critically ill adult patients who met the Berlin Definition criteria for moderate or severe ARDS and required invasive mechanical ventilation within 48 h of ICU admission and for more than 48 h. Clinical course characteristics and key laboratory markers were systematically compared and analyzed according to HLI diagnostic status across all enrolled patients. HLI was defined according to established criteria as elevation of aminotransferase levels (>20-fold upper-limit-of-normal). Predictors of HLI occurrence, clinical course, and outcome were assessed.

**Results:**

In total, 434 critically ill patients with ARDS were included. During the ICU stay, 22.4% (*n*=97) developed HLI. Baseline characteristics including age, sex, and body mass index were comparable between patients with and without HLI. The median Charlson Comorbidity Index was comparable in both groups (3 *vs.* 3 points; *P*=0.36). A higher disease severity on admission represented by the sequential organ failure assessment (SOFA) score was observed in patients with HLI (11 *vs.* 10 points; *P* <0.001). The median arterial partial pressure of oxygen (PaO_2_)/ fraction of inspired oxygen (FiO_2_) ratio was 81 mmHg (interquartile range [IQR]: 60–109 mmHg) in patients with HLI compared to 79 mmHg (IQR: 60–106 mmHg) in patients without HLI, respectively (*P*=0.900). Overall, 35.0% (*n*=152) of patients required veno-venous extracorporeal membrane oxygenation therapy, including 39.2% (*n*=38) within the HLI group and 33.8% (*n*=114) without HLI. The incidence of renal replacement therapy (RRT) was 87.6% (*n*=85) among patients with HLI and 53.1% (*n*=179) among those without HLI (*P* <0.001). ICU mortality was 74.2% (*n*=72) and 58.2% (*n*=196) in the patients with or without HLI, respectively (*P* <0.001). Regression analysis identified cholestasis (odds ratio [OR]=2.061, 95% confidence interval [CI]: 1.220 to 3.481, *P* <0.010) and RRT (OR=4.732, 95% CI: 2.416 to 9.267, *P* <0.001) as factors significantly associated with new-onset HLI. Occurrence of HLI was independently associated with mortality (hazard ratio [HR]=1.710, 95% CI: 1.273 to 2.297, *P* <0.001).

**Conclusions:**

HLI is a common condition in critically ill patients with ARDS, and its occurrence is highly associated with mortality. HLI may serve as prognostic marker in critically ill patients with ARDS.

## Introduction

Acute respiratory distress syndrome (ARDS) is a life-threatening condition frequently encountered in intensive care units (ICUs) worldwide with a significant mortality rate between 35% and 45%.^[^[1], [2], [3]^]^ The pathophysiology of ARDS comprises several aspects, such as elevated permeability of the alveolo-capillary membrane (diffuse alveolar damage, DAD), increase of non-aerated lung-tissue, leading to a higher lung elastance (lower compliance) of the lung and increased dead space, which results in hypoxemia and hypercapnia.^[^^2^^,^^4^^]^

Liver injury and failure are frequently observed types of organ failure during critical illness, and their occurrence is associated with increased morbidity and mortality.^[^[5], [6], [7], [8], [9], [10], [11]^]^ The most commonly observed type of liver injury in critically ill patients is hypoxic liver injury (HLI).^[^^5^^,^^12^^,^^13^^]^ HLI is also known as shock liver, hypoxic hepatitis, or ischemic hepatitis.^[^[14], [15], [16], [17]^]^ The condition of HLI is described as a life-threatening complication defined as a sharp increase of aminotransferase levels accompanied by states of oxygen depletion in an acute setting of cardiac septic or respiratory failure.^[^^14^^,^^15^^]^ Generally, HLI can be found in up to 10% of patients admitted to medical ICUs and its occurrence is associated with mortality rates of over 50%.^[^^5^^,^^12^^]^ Although HLI is most commonly associated with cardiac failure or septic shock, HLI can be observed in the setting of severe hypoxemia in the absence of cardiac and circulatory failure.^[^^9^^,^^11^^,^^14^^,^^18^^,^^19^^]^ Data of critically ill patients with COVID-19 showed that >30% patients suffer from HLI and that the occurrence of HLI was associated with severity of illness.^[^^8^^]^ However, data on the occurrence, course, and outcome of HLI in ARDS is currently scarce.

In the present study, we aimed to investigate the incidence, clinical characteristics, and outcome of HLI in critically ill, mechanically ventilated patients with ARDS in a large tertiary care university hospital.

## Methods

### Study design and setting

This retrospective single-center cohort study was conducted at the Department of Intensive Care Medicine at the University Medical Center Hamburg-Eppendorf, which is a tertiary care university hospital in northern Germany. The department comprises 12 ICUs with a total capacity of 100–120 beds, providing care for critically ill adult patients. We retrospectively included all adult patients admitted to the ICUs of our department who met the Berlin criteria for moderate-to-severe ARDS between December 1, 2017 and April 30, 2021. Inclusion required invasive mechanical ventilation within 48 h of ICU admission and for more than48 h. Patients receiving only high-flow nasal oxygen or non-invasive ventilation without intubation, or developing ARDS after 48 h of ICU admission, were excluded.

This study was approved by the Ethics Committee of the Hamburg Chamber of Physicians (No. 2023-300318-WF). The need for consent was waived due to the retrospective character of the study and the anonymization of data upon retrieval.

### Clinical definitions and patients management

HLI was defined according to established criteria as an elevation of aminotransferase levels (>20-fold upper limit of normal) and divided the patients into two groups (HLI group *vs*. no HLI group).^[^^5^^,^^7^^,^^17^^]^ Cholestasis was defined as rise of total bilirubin ≥3 mg/dL within the ICU stay.^[^^6^^,^^17^^]^ ARDS was defined on the basis of the Berlin definition, with arterial patiral pressure of oxygen (PaO_2_)/ fraction of inspired oxygen (FiO_2_(partial arterial pressure of oxygen/fraction of inspired oxygen) indicating the severity of the disease.^[^^3^^]^ Clinical management was realized in accordance with national and international guidelines, such as restrictive fluid management following the initial resuscitation period, lung-protective ventilation using pressure-controlled ventilation mode (biphasic positive airway pressure), administration of inhalational nitric oxide, and prone positioning in moderate to severe ARDS.^[^^20^^]^ Lung-protective ventilation strategies were performed with a maximum tidal volume (V_T_) of 6 mL/kg of predicted body weight, permissive hypercapnia with pH ≥ 7.2 and positive end-expiratory pressure (PEEP) titration according to the ARDS network recommendation with a maximum ΔP of 1500 Pa.^[^^2^^]^ Patients with severe ARDS, refractory to adjunctive measures, received veno-venous extracorporeal membrane oxygenation (VV-ECMO). Criteria for the initiation of extracorporeal life support were defined according to the guidelines of the Extracorporeal Life Support Organization and national recommendations.^[^[20], [21], [22]^]^ Severe acute kidney injury (AKI) was defined according to the international KDIGO (Kidney Disease: Improving Global Outcomes) guidelines.^[^^23^^]^ Criteria for the initiation of renal replacement therapy (RRT) were defined according to the standard operating procedure of our institution and in accordance with national recommendations.^[^^24^^,^^25^^]^ Vasopressor support was initiated to obtain a mean arterial pressure of 65 mmHg using norepinephrine.^[^^20^^,^^26^^]^

### Data collection

All data were extracted from the information system routinely used at our institution (Integrated Care Manager [ICM], Version 9.1 - Draeger Medical, Lübeck, Germany). The data included sex, age, body mass index, comorbidities, admission diagnosis, length of hospital stay, organ support (mechanical ventilation, prone positioning, ECMO, vasopressor support, RRT), medication, blood gas analyses, and laboratory test results. Survival was assessed at ICU discharge.

### Statistical analysis

Data are presented as absolute numbers and relative frequency or median with interquartile range (IQR) depending on the distribution of the data. Categorical variables were compared via Chi-squared analysis or Fisher’s exact test, as appropriate. Continuous variables were compared via Mann–Whitney *U* test. The distribution of variables was graphically assessed with histograms. Spearman correlation coefficients were calculated to evaluate potential collinearity. We clinically assessed factors based on their clinical relevance associated with the occurrence of HLI and mortality. We used multivariable logistic regression with HLI as the dependent variable and relevant clinical factors as independent covariables. In addition, a multivariable Cox proportional hazards model was applied to estimate the effect of HLI on survival. In both models, we used forced entry approach. Statistical analysis was conducted using IBM SPSS Statistics Version 24.0 (IBM Corp., Armonk, New York). Generally, a *P* value <0.05 was considered statistically significant. The study was prepared in accordance with the STrengthening the Reporting of OBservational studies in Epidemiology recommendations.^[^^27^^]^

## Results

### Study population

During the study period from December 1, 2017, to April 30, 2021, a total number of 1519 patients who received mechanical ventilation within the first 48 h after ICU admission were identified. Of those, 1079 did not fulfil the Berlin criteria for ARDS, additional five patients had a PaO_2_/FiO_2_ between 200 and 300 mmHg (mild ARDS) and were therefore excluded from the study. After excluding one more patient due to incomplete data, 434 patients were included in the final analysis (Figure 1).

**Figure 1:**
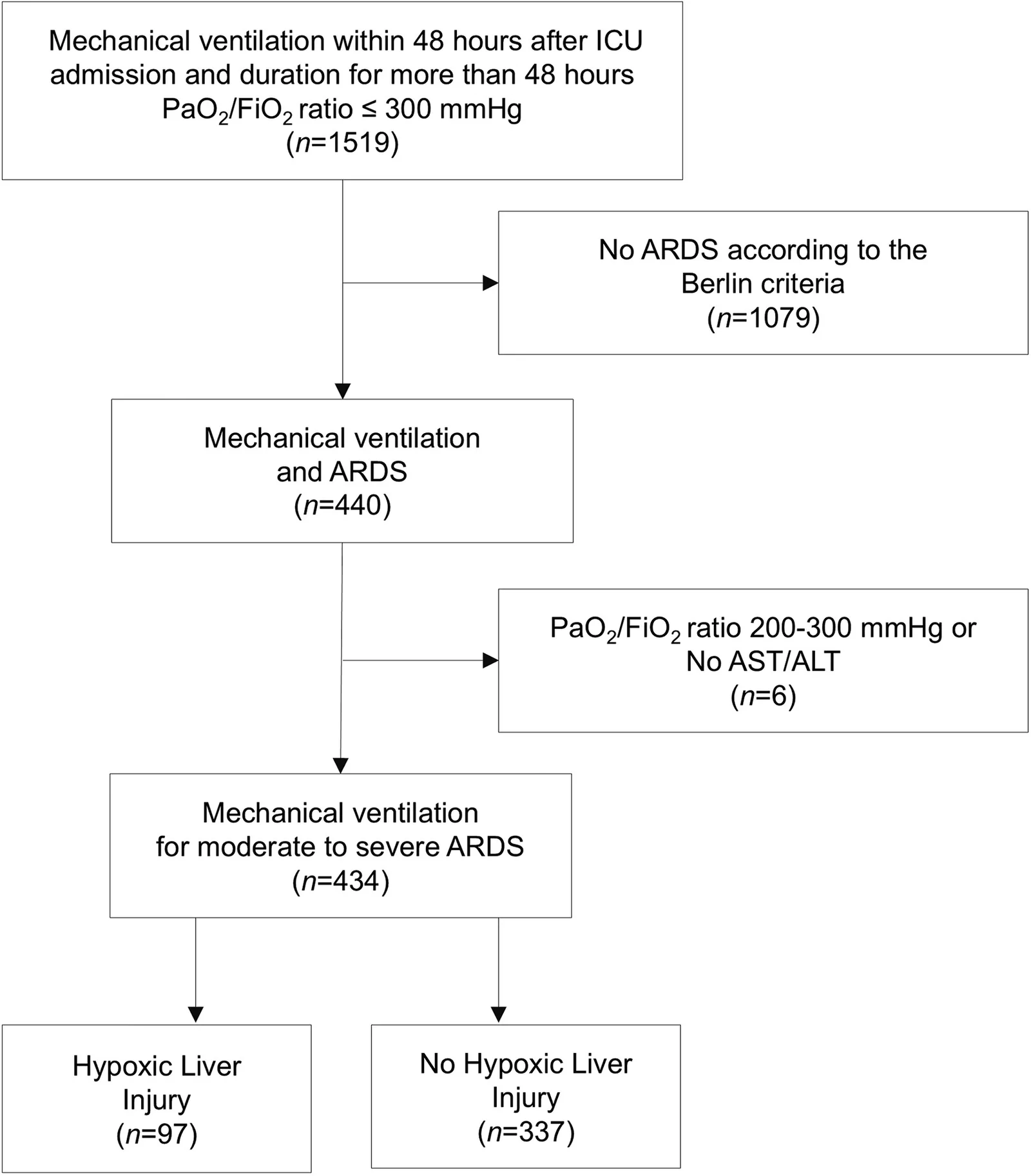
Flow chart of the study. ARDS, Acute respiratory distress syndrome; ALT, Alanine aminotransferase; AST, Aspartate aminotransferase; ICU, Intensive care unit; PaO2/FiO2, Arterial partial pressure of oxygen/fraction of inspired oxygen.

### Baseline demographic characteristics

Baseline demographic characteristics are reported in Table 1. The median age of patients was 59 (IQR: 49–69) years, 67.9% (*n*=295) were male and the median BMI was 27.5 (IQR: 24.2–32.8) kg/m^2^. The median Charlson Comorbidity Index (CCI) was 3 (IQR: 2–5) points. The most common comorbidities were arterial hypertension (46.5%, *n*=202), diabetes mellitus type Ⅱ (21.4%, *n*= 93), and coronary heart disease (11.5%, *n*=50). The median duration of ICU stay was 20.1 (IQR: 11.4–33.3) days, respectively.

**Table 1:** Baseline characteristics of the study population (*n*=434).

Variables	Data
Age (years)	59 (49–69)
Males	295 (67.9)
BMI (kg/m²)	27.5 (24.2–32.8)
Comorbidities	
Charlson comorbidity Index (points)	3 (2–5)
Arterial hypertension	202 (46.5)
Chronic kidney disease	24 (5.5)
Coronary heart disease	50 (11.5)
Diabetes mellitus	93 (21.4)
Chronic lung disease	49 (11.3)
Liver disease	39 (9.0)
ARDS etiology	
Pneumonia	368 (84.8)
Extrapulmonary sepsis	11 (2.5)
Aspiration	42 (9.7)
Noncardiogenic shock	4 (0.9)
Pulmonary contusion	2 (0.5)
Inhalation	1 (0.2)
Pulmonary vasculitis	1 (0.2)
Pancreatitis	1 (0.2)
Transfusion	2 (0.5)
Others	2 (0.5)
Pneumonia etiology	
Community-acquired pneumonia	255 (58.8)
Hospital-acquired pneumonia	32 (7.4)
Pneumonia in immunocompromised patients	79 (18.2)
COVID-19	12 (2.8)
Influenza	24 (5.5)

Data are expressed as *n* (%) or median (interquartile range).

ARDS, Acute respiratory distress syndrome; BMI, Body mass index; ICU, Intensive care unit.

### Occurrence and clinical characteristics of patients with and without HLI

Of the total study cohort (*n*=434), 22.4% (*n*=97) developed HLI during the ICU stay. The diagnostic criteria for HLI were met median of 3 (IQR: 1–14) days after ICU admission. The highest median aspartate aminotransferase (AST) was 2028 (IQR: 850 – 6671) U/L and the highest median alanine aminotransferase (ALT) was 843 (IQR: 380–1917) U/L. The peak aminotransferase level was recorded median of 4 (IQR: 1–17) days after ICU admission. In the study cohort, 46.3% (*n*=201) of the patients suffered from cholestasis (≥3 mg/dL) newly developed during the ICU stay, 66.0% (*n*=64) of patients within the HLI group suffered from cholestasis.

Detailed ICU characteristics of patients in HLI group and non-HLI group are shown in Table 2. Patients in HLI group presented with higher disease severity, represented by the sequential organ failure assessment score (SOFA) (11 *vs*. 10 points; *P* <0.001) on admission. The median CCI was 3 (IQR: 2–5) points in HLI group and 3 (IQR: 2–5) in the non-HLI group (*P*=0.360). Twenty-four patients (5.5%) had pre-existing chronic kidney disease; there was no difference between the two groups (*P*=0.410). Liver cirrhosis was present in 9.0% (*n*=39) of the overall cohort; the prevalence was significantly higher in HLI group than in non-HLI group (17.5% *vs.* 6.5%; *P* <0.001) (Supplementary Table S1).

**Table 2:** ICU characteristics of patients with and without hypoxic liver injury.

Variables	All patients (*n*=434)	HLI group (*n*=97)	Non-HLI group (*n*=337)	*P* value
Age (years)	59 (49–69)	60 (49–70)	59 (49–69)	1.000
Males	294 (67.7)	65 (67.0)	229 (68.0)	0.860
BMI (kg/m²)	27.5 (24.2–32.8)	27.8 (24.7–33.5)	27.3 (24.1–32.5)	0.250
Disease Severity				
SOFA – admission (points)	10 (8–12)	11 (9–13)	10 (8–12)	<0.001
Charlson Comorbidity index (points)	3 (2–5)	3 (2–5)	3 (2–5)	0.360
Respiratory function/support				
PaO_2_ (mmHg)	57.8 (51.8–63.7)	57.4 (50.9–65.5)	57.8 (52.0–63.6)	0.890
FiO_2_ (%)	80 (56–100)	88 (60–100)	75 (53–100)	<0.050
PaO_2_/FiO_2_ (mmHg)	79 (60–106)	81 (60–109)	79 (60–106)	0.900
Invasive MV	434 (100)	97 (100)	337 (100)	1.000
Duration of MV (days)	16.7 (8.2–29.6)	15.7 (6.3–30.7)	16.8 (8.9–29.3)	0.250
PEEP (Pa)	1100 (900–1500)	1200 (900–1500)	1100 (900–1400)	0.410
Respiratory Rate	22 (18–26)	23 (18–26)	22 (18–26)	0.550
Inspiratory pressure (Pa)	2500 (1900–3000)	2500 (2000–3000)	2500 (1900–3000)	0.950
VV-ECMO	152 (35.0)	38 (39.2)	114 (33.8)	0.330
Inhaled Nitric Oxide	179 (41.3)	53 (54.6)	126 (37.4)	<0.010
Prone Positioning	285 (66.7)	66 (68.0)	219 (65.0)	0.580
Driving Pressure (Pa)	1500 (1200–1800)	1500 (1200–1800)	1500 (1200–1800)	0.850
Tidal Volume (mL)	419 (331–505)	437 (351–528)	414 (326–504)	0.150
Hemodynamic data (mmHg)				
MAP on admission	77 (70–87)	77 (70–88)	78 (65–86)	0.260
MAP after 24 h	73 (67–78)	73 (67–79)	79 (65–76)	0.420
MAP after 48 h	73 (68–79)	73 (68–80)	72 (67–77)	0.350
MAP after 72 h	73 (68–80)	74 (69–80)	71 (65–77)	0.330
Vasopressor use (µg/(kg·min))				
Norepinephrine on admission	0.25 (0.08–0.60)	0.23 (0.08 – 0.50)	0.45 (0.14–0.81)	0.820
Norepinephrine after 24 h	0.24 (0.09–0.45)	0.22 (0.08 – 0.37)	0.42 (0.17–0.67)	0.950
Norepinephrine after 48 h	0.19 (0.08–0.44)	0.16 (0.07 – 0.35)	0.37 (0.12–0.72)	0.290
Norepinephrine after 72 h	0.15 (0.06–0.34)	0.12 (0.05 – 0.29)	0.31 (0.13–0.52)	0.570
ARDS etiology				<0.001
Pulmonary	417 (96.1)	88 (90.7)	329 (97.6)	
Extrapulmonary	14 (3.2)	6 (6.2)	8 (2.4)	
None	2 (0.5)	2 (2.1)	0 (0)	
Both	1 (0.2)	1 (1.0)	0 (0)	
Pneumonia etiology				0.060
Community-acquired pneumonia	255 (58.8)	54 (55.7)	201 (59.6)	
Hospital-acquired pneumonia	32 (7.4)	5 (5.2)	27 (8.0)	
Pneumonia in immunocompromised patients	79 (18.2)	19 (19.6)	60 (17.8)	
COVID-19	127 (29.3)	27 (27.8)	100 (29.7)	0.730
Influenza	24 (5.5)	8 (8.2)	16 (4.7)	0.180
Procedures/Therapies				
Renal replacement therapy	264 (60.1)	85 (87.6)	179 (53.1)	<0.001
Fluid balance (24 h) (mL)	1978 (800–3724)	2859 (920–5745)	1846 (655–3355)	<0.001
Laboratory results				
AST – peak (U/L)	195 (109–534)	2028 (850–6671)	154 (95–242)	<0.001
ALT – peak (U/L)	129 (65–304)	843 (380–1917)	96 (57–156)	<0.001
Bilirubin – peak (mg/dL)	2.8 (1.6–6.0)	4.2 (2.6–12.4)	2.4 (1.5–4.4)	<0.001
Bilirubin > 3 mg/dL	201 (46.3)	64 (66.0)	137 (40.7)	<0.001
Outcome				
Duration of ICU stay (days)	20.1 (11.4–33.3)	18.2 (7.8–31.9)	20.2 (12.3–33.8)	<0.050
Died in ICU	213 (49.1)	72 (74.2)	196 (58.2)	<0.001

Data are expressed as *n* (%) or median (interquartile range).

ALT, Alanine aminotransferase; ARDS, Acute respiratory distress syndrome; AST, Aspartate aminotransferase; BMI, Body mass index; ECMO, Extracorporeal membrane oxygenation; ICU, Intensive care unit; LDH, Lactate-dehydrogenase; PEEP, Positive end-expiratory pressure; MAP, Mean arterial pressure; SOFA, Sequential organ failure assessment.

The baseline PaO_2_ was comparable in both groups. The median PaO₂/FiO₂ ratio did not differ significantly between the HLI and non-HLI groups; however, the median FiO₂ was significantly higher in the HLI group than in the non-HLI group (88% [IQR: 60–100] *vs*. 75% [IQR: 53–100]; *P* <0.050). Detailed respiratory function, hemodynamic data, and vasopressor use in patients with and without HLI are presented in Table 2.

Adjunctive treatment including prone positioning (68.0 *vs*. 65.0%; *P*=0.58) and inhaled nitric oxide (54.6% *vs*. 37.4%) were frequent in HLI group and non-HLI group (*P* <0.010). Overall, 35.0% (*n*=152) of patients required VV-ECMO therapy; of these, 39.2% (*n*=38) were from HLI group and 33.8% (*n*=114) were from the non-HLI group. The median duration of mechanical ventilation was 15.7 (IQR: 6.3–30.7) days in HLI group and 16.8 (IQR: 8.9–29.3) days in non-HLI group (*P*=0.25). All patients received vasopressor therapy. The cause of ARDS was primarily pulmonary in both groups (HLI group: 90.7% *vs*. non-HLI group 97.6%). The most common pulmonary etiology was community-acquired pneumonia, present in 55.7% (*n*=54) of patients in the HLI group and 59.6% (*n*=201) of those in the non-HLI group. The incidence of RRT was significantly higher in the HLI group (87.6%, *n*=85) than in the non-HLI group (53.1%, *n*=179; *P* <0.001). Median cumulative fluid balance within the first 24 h was also significantly greater in the HLI group (+2859 mL *vs*. +1846 mL; *P* <0.001).

### Outcomes of critically ill patients with and without HLI

Overall, an ICU mortality rate of 49.1% (*n*=213) was observed. The ICU mortality was 74.2% (*n*=72) and 58.2% (*n*=196) in patients with HLI compared to patients without HLI, respectively (*P* <0.001). The median length of stay at the ICU was 18.2 (IQR: 7.8–31.9) and 20.2 (IQR: 12.3–33.8) days in patients with and without HLI (*P* <0.050), respectively.

### Risk factors for HLI and mortality

Multivariable regression analysis identified cholestasis (OR=2.061, 95% CI: 1.220 to 3.481; *P* <0.010) and presence of RRT (OR=4.732, 95% CI: 2.416 to 9.267; *P* <0.001) as factors significantly associated with new-onset HLI (Table 3). After adjustment for confounders, we observed that HLI (HR=1.710, 95% CI: 1.273 to 2.297; *P* <0.001) and RRT (OR=1.970, 95% CI: 1.395 to 2.783; *P* <0.001) were significantly associated with ICU mortality (Table 4).

**Table 3:** Multivariable logistic regression for factors associated with occurrence of hypoxic liver injury.

Covariables	OR (95% CI)	*P* value
Sex (male *vs.* female)	0.727 (0.423–1.250)	0.250
BMI (points)⁎	1.020 (0.994–1.047)	0.140
CCI (points)*	1.110 (0.997–1.235)	0.060
Renal replacement therapy (yes *vs.* no)	4.732 (2.416–9.267)	<0.001
Cholestasis (yes *vs.* no)	2.061 (1.220–3.481)	<0.010
Driving pressure (level)	0.959 (0.904–1.016)	0.160

CI, Confidence interval; CCI, Charlson comorbidity index; HLI, Hypoxic liver injury; OR, Odds ratio; SAPS II, Simplified acute physiology score II.

⁎ CCI and, BMI were transformed prior to logistic regression analysis (natural logarithm).

**Table 4:** Multivariable Cox regression analysis for factors associated with mortality.

Covariables	HR (95% CI)	*P* value
HLI (yes *vs.* no)	1.710 (1.273–2.297)	<0.001
Sex (male *vs.* female)	0.752 (0.555–1.021)	0.070
CCI (points)*	1.012 (0.952–1.075	0.710
BMI (points)*	0.987 (0.972–1.003)	0.110
Driving pressure (level)*	1.002 (0.973–1.033)	0.900
Renal replacement therapy (yes *vs.* no)	1.970 (1.395–2.783)	<0.001
SOFA score (Points)	1.033 (0.988–1.081)	0.150

BMI, Body mass index; CI, Confidence interval; CCI, Charlson comorbidity index; HR, Hazard ratio; HLI, Hypoxic liver injury; SOFA, Sequential organ failure assessment.

⁎ CCI, BMI, and driving pressure on admission were transformed prior to logistic regression analysis (natural logarithm).

## Discussion

This retrospective study showed that HLI affects 22% of patients with moderate to severe ARDS and is therefore a common complication. Furthermore, its occurrence was associated with a significantly higher mortality compared to ARDS patients without HLI. To our knowledge, few studies have examined the incidence and outcomes of HLI in patients with ARDS.

In general, ARDS is a life-threatening condition with a mortality rate reaching up to 45% in its severe form, and it accounts for about 10% of admissions to ICUs and for around 23% of all ventilated patients.^[^^2^^,^^3^^]^ Liver injury and failure are frequently observed types of organ failure during critical illness, and their occurrence is associated with increased morbidity and mortality.^[^[5], [6], [7], [8], [9], [10], [11]^]^ Secondary or acquired liver injury and failure such as HLI or cholestatic dysfunction in critical illness represent by far the most frequent forms of hepatic injury in critical illness.^[^^5^^,^^12^^,^^13^^,^^17^^]^ Reports in patients with ARDS showed that early hepatic dysfunction defined as cholestasis is associated with worse outcome.^[^^28^^]^ However, regarding HLI, the data is scarce. HLI is a life-threatening condition occurring in the setting of cardiac, circulatory, or respiratory failure.^[^^29^^,^^30^^]^ In mixed ICU populations, HLI occurs in up to 10% of patients and reaches a mortality rate of up to 50%.^[^^5^^,^^31^^,^^32^^]^ Most cases of HLI are associated with cardiac failure or septic shock and only few reports describe the occurrence of HLI associated with severe respiratory failure.^[^^33^^]^ Several studies characterize the pathogenesis of HLI as multifactorial, with all origins of the disease merging in a common pathway, which is hepatocellular dysfunction due to critically low levels of oxygen for metabolic function.^[^^15^^,^^34^^]^ Since we only included patients with moderate to severe ARDS (PaO_2_/FiO_2_ < 200 mmHg), our study cohort naturally suffered from hypoxemia, which most likely affected the hepatocytes. Thus, our study found a substantially high number of HLI of 22% in patients with ARDS, which is around 2-fold higher than described in other large primary mixed ICU cohorts.^[^^5^^,^^31^^]^ Similar PaO_2_/FiO_2_ ratios between HLI and non-HLI groups suggest that HLI does not necessarily reflect greater severity of pulmonary dysfunction. Instead, the higher prevalence of AKI requiring RRT among patients with HLI in this cohort supports the interpretation that HLI is a marker of systemic hypoxia–ischemia and multi-organ dysfunction. Thus, the association between HLI and mortality likely reflects overall illness severity rather than a direct causal effect on ARDS progression. Therefore, our findings are in line with earlier studies that describe HLI as being multifactorially triggered in >70% of cases.^[^^35^^]^

Remarkably, our results indicate a higher mortality among patients with both HLI and ARDS (74%) compared with previously reported mortality rates in patients with either HLI or ARDS (50% and 45%).^[^^2^^,^^32^^]^ To our knowledge, this is the first study investigating the occurrence of HLI in patients with moderate to severe ARDS. However, research by Roedl et al.^[^^8^^]^ showed a high incidence of HLI in association with increased mortality in critically ill patients with severe COVID-19. As this study only included patients with COVID-19 and used a different cut-off for the diagnosis of severe liver dysfunction, it is unclear whether the results can be compared to other causes of respiratory failure (non-COVID) and moderate to severe ARDS. Furthermore, it remains unclear whether COVID-19 may have a direct viral effect on the liver, which may not occur in other disease entities.^[^^36^^]^ Since we found a distinct increase of mortality in patients with HLI and ARDS, it is crucial to compare the mortality of our study population to different cohorts of patients with HLI. For instance, one study found that the mortality in patients with HLI after cardiopulmonary resuscitation was 72.2% (*vs*. 54.9% in the non-HLI group), which is similar to the mortality that we found in our study cohort.^[^^37^^,^^38^^]^ On the other hand, Jung et al.^[^^38^^]^ investigated the mortality of patients with HLI in cardiogenic shock and found a significant increase in 30-day mortality in patients with HLI compared to patients without HLI (68% *vs*. 34%, *P* <0.001). These results are consistent with our findings regarding mortality in patients with HLI. Remarkably, however, mortality in our study cohort was still higher than in patients with cardiogenic shock, raising the question of the underlying reasons for this observation.

The increase in mortality among patients with both HLI and ARDS, compared with those with either HLI or ARDS, highlights the complex interaction between the lung and the liver. Recent evidence suggests that the liver is a crucial factor in the pathogenesis and resolution or ARDS.^[^^4^^]^ Although the mechanisms of liver-lung interaction in the pathogenesis of ARDS are not completely understood, it is evident that some functions of the liver, such as the release and clearance of proinflammatory cytokines and eicosanoids, the clearance of bacteria and endotoxins and also the synthesis of acute-phase proteins, can modulate lung injury in the presence of severe inflammatory disease or sepsis.^[^^4^^]^ Hence, our findings may suggest that the presence of HLI further aggravates ARDS, resulting in higher mortality in this group of patients. Aside from the complex interaction between the liver and the lung, the increase in mortality in ARDS patients with HLI might possibly occur due to the severity of the underlying disease. This assumption is supported by a previous study from Roedl et al.,^[^^8^^]^ who identified a higher median SAPS II (Simplified Acute Physiology Score II) and SOFA score on admission in COVID-19 patients, who developed liver injury during the ICU stay. In our study, we found a slightly higher SOFA score in the HLI group compared to the patients without HLI. Although we found only a slight difference regarding SOFA, this could partially explain increased mortality among patients with HLI. Furthermore, we observed more patients with underlying liver cirrhosis in the HLI group. This is of special interest because patients with liver cirrhosis and ARDS suffer from an increased mortality.^[^^39^^,^^40^^]^ It was highlighted that early recognition and treatment for infection might be important for improving outcomes.

Although HLI presents as a heterogenous condition, studies have identified risk factors that are associated with the development of HLI. Fuhrmann et al.^[^^5^^]^ described cardiomyopathy, RRT, and vasopressor therapy as the most prominent factors that are associated with HLI, whereas Drolz et al.^[^^12^^]^ identified AKI as one main risk factor. In our study, we found that HLI was significantly associated with the occurrence of AKI and need for RRT. Therefore, the results of our study were consistent with previous studies. Furthermore, it is worth noting that our results showed a difference in fluid balance between the two groups within the first 24 h, the HLI cohort being the group with a higher fluid balance compared to the group of patients without HLI. This may be a critical insight, since congestive heart failure was identified as a risk factor for the development of HLI.^[^^31^^,^^34^^,^^41^^]^ Although we did not find a difference in the occurrence of congestive heart failure between the HLI group and the non-HLI group, the pathophysiology of fluid overload and congestive heart failure remains similar. However, a higher SOFA score could also be derived from a high rate of sepsis in the cohort, which often comes along with high fluid intake. Another interesting finding of our study was a slight difference in PEEP between the ARDS patients with HLI compared to the group without HLI (1200 *vs*. 1100 Pa). Although the difference was not statistically significant, it is worth mentioning because higher PEEP titration (≥1000 Pa) may in fact be detrimental to liver function.^[^^42^^,^^43^^]^ This becomes particularly important in moderate to severe ARDS as international guidelines recommend increased PEEP up to 2400 Pa in severe hypoxemia.^[^^2^^]^

Compared to large observational ARDS cohorts (e.g., Lung Safe) the median ICU length of stay was prolonged.^[^^1^^]^ This difference likely reflects the selected nature of our population, which consisted of patients with moderate-to-severe ARDS treated at tertiary referral centers and requiring prolonged mechanical ventilation. In addition, as a specialized center, we frequently receive complex referrals, including patients transferred after clinical deterioration at outside hospitals. This may contribute to longer ICU courses. Furthermore, differences in healthcare systems, step-down unit availability, and rehabilitation pathways may also influence ICU discharge timing and prolong ICU stay.

### Strengths and limitations

Our study has several limitations, the first one being the study design. Since our study was a retrospective analysis, there may exist unmeasured confounding factors that may have affected the outcome of interest. Also, the results of our study only apply to HLI patients with moderate-to-severe ARDS, and the results cannot be applied to other cohorts of patients with HLI. Given the retrospective observational design, the associations between HLI, RRT use, and ICU mortality cannot be interpreted as causal and may reflect underlying disease severity and multi-organ dysfunction. We observed differences in the development of HLI in patients with and without liver cirrhosis. However, conclusions regarding this finding should be done cautiously, as we do not have more information about the underlying origin of liver cirrhosis and disease severity. Due to the retrospective design, locked database structure, and ethical approval, longer-term outcomes such as 28- or 90-day mortality could not be assessed, restricting outcome evaluation to ICU mortality and ICU length of stay.

The most important strength of our retrospective cohort study is the data set that we used. The data of all patients were meticulously screened and only patients with moderate to severe ARDS according to the Berlin criteria, who required mechanical ventilation within the first 48 h after ICU admission for a duration of >48 h were included in the study.^[^^2^^]^ This homogenous data set drastically improves the reliability of our results.

## Conclusions

HLI is a common condition in critically ill patients with ARDS, which affects >20% of patients with moderate to severe ARDS. Occurrence of HLI is associated with high mortality. Therefore, HLI may serve as a prognostic marker in critically ill patients.

## CRediT authorship contribution statement

**Fabian Gleibs:** Writing – original draft, Validation, Methodology, Investigation, Data curation. **Josephine Braunsteiner:** Validation, Methodology, Investigation, Formal analysis, Data curation. **Markus Haar:** Software, Resources, Project administration, Methodology, Investigation, Conceptualization. **Stefan Kluge:** Validation, Supervision. **Marlene Fischer:** Validation, Supervision, Resources, Project administration, Methodology, Investigation, Formal analysis, Conceptualization. **Kevin Roedl:** Validation, Supervision, Funding acquisition, Formal analysis, Conceptualization.

## Appendix Supplementary Materials

Image, application 1
