# Supplementary material for: Occurrence of hypoxic liver injury in patients with acute respiratory distress syndrome: A retrospective cohort study
Source: J Intensive Med. 2026 May 9;6(5):501–8. doi: 10.1016/j.jointm.2026.02.006 (PMC13728385; doi:10.1016/j.jointm.2026.02.006)
Supplement: Supplementary file 1 [file mmc1.docx]

*Supplementary Table S1 – Pre-existing comorbidities*

| *Parameters* | *All*  *(n = 434)* | *Hypoxic liver injury*  *(n = 97)* | *No – Hypoxic liver injury (n = 337)* | *P*-value |
| --- | --- | --- | --- | --- |
| AIDS | 6 (1.4) | 3 (3.1) | 3 (0.9) | 0.13 |
| Cerebral arterial disease | 37 (8.5) | 9 (9.3) | 28 (8.3) | 0.76 |
| Chronic lung disease | 49 (11.2) | 10 (10.3) | 39 (11.6) | 0.73 |
| Chronic kidney disease | 24 (5.5) | 7 (7.2) | 17 (5.0) | 0.41 |
| Congestive heart disease | 48 (11.0) | 10 (10.3) | 38 (11.3) | 0.79 |
| Connective tissue disease | 32 (7.4) | 5 (5.1) | 27 (8.0) | 0.34 |
| Coronary heart disease | 50 (11.5) | 13 (13.4) | 37 (11.0) | 0.51 |
| Dementia | 8 (1.8) | 2 (2.1) | 6 (1.8) | 0.86 |
| Diabetes Mellitus | 93 (21.4) | 23 (23.7) | 70 (20.8) | 0.26 |
| Liver cirrhosis | 39 (9.0) | 17 (17.5) | 22 (6.5) | < 0.001 |
| Peripheral arterial disease | 24 (5.5) | 4 (4.1) | 20 (5.9) | 0.49 |
| Malignancy *n*  Solid tumor  Leukemia  Lymphoma  Solid tumor with metastases | 49 (11.3)  25 (5.8)  17 (3.9)  12 (2.8) | 10 (10.3)  5 (5.2)  4 (4.1)  3 (3.1) | 39 (11.6)  20 (5.9)  13 (3.9)  9 (2.7) | 0.92  0.77  0.91  0.92 |

*Abbreviations: AIDS, acquired immune deficiency syndrome; n, number;*
